# Urgent and emergent breast lesions – A primer for the general radiologist, on‐call resident and sonographer

**DOI:** 10.1002/ajum.12296

**Published:** 2022-05-12

**Authors:** Asha A. Bhatt, Genevieve A. Woodard, Christine U. Lee, Gina K. Hesley

**Affiliations:** ^1^ Department of Radiology Mayo Clinic 200 1^st^ Street SW Rochester Minnesota 55905 USA

**Keywords:** breast abscess, breast emergencies, breast necrotising fasciitis, breast pseudoaneurysm, mastitis, nipple piercings

## Abstract

There are very few true breast emergencies. While infrequent, women do present to emergency departments or urgent care centres with breast‐related concerns. In this case‐based review, both common and uncommon urgent and emergent breast lesions are presented, emphasising ultrasound characteristics and imaging optimisation to improve accurate diagnosis and appropriate recommendations.

## Introduction

Ultrasound is a modality that is readily available at a low cost and does not use ionising radiation, making it ideal for evaluating patients with breast‐related complaints in an urgent or emergent setting.^1^ When evaluating the breast in these settings, the primary goal is to efficiently identify the acute cause for the patient's presenting symptoms. This is in contrast to when ultrasound is utilised during a diagnostic imaging evaluation where the rationale is to identify or exclude a malignancy as the aetiology for a patient's presentation. This article reviews the most common causes for breast‐related urgent and emergent complaints, including trauma, bleeding and infection. Emphasis is placed on salient ultrasound imaging features to make an accurate diagnosis of a benign process and aid the recognition of a possible underlying malignancy that would require additional diagnostic imaging and biopsy. This review was approved by the Institutional Review Board of our institution's Office for Human Research Protections.

At our institution and at many imaging centres across the country, targeted breast ultrasound examinations performed after‐hours are not always interpreted by a *Mammography Quality Standards Act* (MQSA)‐certified breast radiologist. Furthermore, imaging reports often contain a disclaimer such as ‘This focused breast ultrasound examination was performed to evaluate for infection and fluid collections only. To assess for malignancy, a full diagnostic workup in the Breast Imaging department would be necessary’. American College of Radiology (ACR) Breast Imaging‐Reporting and Data System (BI‐RADS) assessments (Table 1) are not used when generating a report for these cases; therefore, these cases are not included in an MQSA audit.^2^ Given the infrequency of these after‐hours cases and the non‐breast subspecialised experience of the interpreting radiologist, consultation with an MQSA‐certified breast radiologist the next day or follow‐up with a dedicated breast imaging evaluation is a common practice.

**Table 1:** ACR BI‐RADS assessment categories.^2^ For each category, the recommended management and likelihood of cancer are shown

ACR BI‐RADS assessment categories			
Category		Management	Likelihood of cancer
0	Needs additional imaging	Obtain additional imaging	n/a
1	Negative	Routine screening	Essentially 0%
2	Benign	Routine screening	Essentially 0%
3	Probably benign	Short‐interval follow‐up	>0% but ≤2%
4	Suspicious	Biopsy	>2% but <95%
5	Highly suggestive of malignancy	Biopsy	≥95%
6	Biopsy‐proven malignancy	Surgical/clinical management	Known cancer

Abbreviation: ACR BI‐RADS, American College of Radiology Breast Imaging‐Reporting and Data System.

Linear ultrasound transducers ranging from 5 to 18 MHz are most commonly used for targeted breast ultrasound.^3^ According to ACR practice guidelines, due to concerns of poor spatial resolution at lower frequencies, the lowest frequency considered ‘allowable’ in breast imaging is a centre frequency of 12 MHz.^4^, ^5^ However, curved or lower frequency linear transducers, commonly utilised for body ultrasound, may be used after‐hours by the on‐call sonographer or radiologist for imaging the breast, and this is another reason patients should be encouraged to pursue dedicated breast imaging evaluation in follow‐up.

## Case‐based review

### Infection

**Figure 1:**
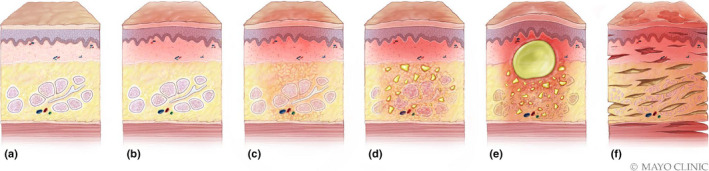
Stages of breast infection: Normal breast tissue and overlying skin (a). Cellulitis is the early mild form of a breast infection, which only involves the dermis (b). Subsequent progression to mastitis results in inflammation of the breast parenchyma and lobules (c). Continued progression can involve microabscesses and phlegmonous change (d), which can form an organised abscess (e). The last possible stage in a breast infection, often associated with diabetes, is necrotising fasciitis, a gas‐forming infection that traverses into multiple facial planes (f). Source: Used with permission of Mayo Foundation for Medical Education and Research, all rights reserved.

Breast infections have a broad spectrum of imaging presentations depending on the severity. The various stages of an infectious process involving the breast are depicted in Figure 1.

**Figure 2:**
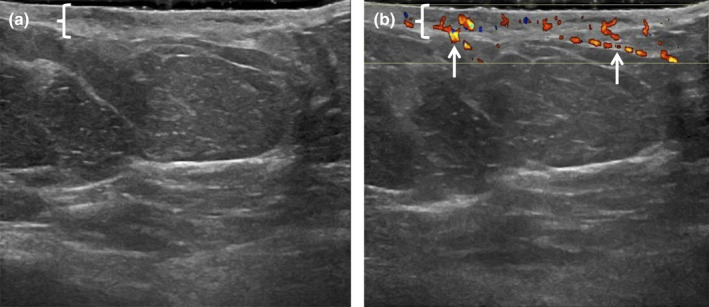
30‐year‐old woman presented with redness and tenderness in the lower central left breast. She was evaluated and started on Bactrim double‐strength twice daily. Subsequently, the patient noticed purulent drainage from the skin with some improvement in her symptoms. The breast ultrasound was performed with a ML6‐15 MHz transducer with a frequency set to 11 MHz. The images were captured from the skin to the chest wall. Targeted ultrasound at the site of focal erythema demonstrated mild skin thickening measuring 4 mm (bracket) with heterogeneous increased echogenicity seen with oedema (a), and associated increased vascularity (b, arrows). No underlying breast findings were observed. Imaging findings are consistent with focal left breast cellulitis without an underlying abscess. Clinical follow‐up of the left breast erythema with repeat targeted imaging in the setting of worsening symptoms was recommended.

Typically, these patients will complain of breast pain and physical examinations reveal localised erythema and swelling or even a palpable lump.^6^ Early infections are localised to the dermis, with isolated skin thickening (>2 mm) that can be appreciated on targeted breast ultrasound examination, confirming the diagnosis of breast cellulitis (Figure 2). The role of ultrasound is to evaluate the progression of infection into the breast parenchyma. Therefore, it is important that ultrasound images should be taken from the skin to the chest wall for these evaluations as to not miss deeper infections.

**Figure 3:**
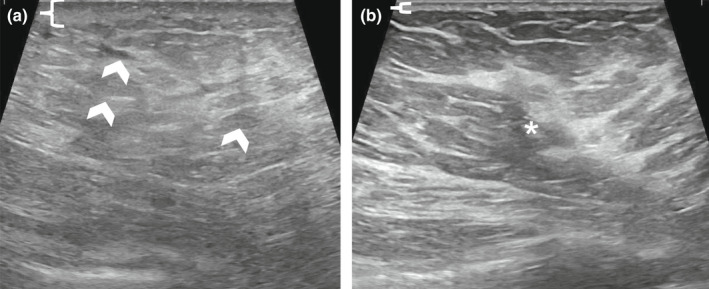
42‐year‐old woman with a history of breast conservation therapy performed 9 months prior presented to the emergency department with worsening right breast pain and redness despite being treated empirically with clindamycin. In the upper outer right breast at the site of clinical concern, ultrasound was performed with an L3‐12 MHz transducer with harmonics and the images were captured from the skin to the chest wall. Targeted ultrasound demonstrated skin thickening measuring 6 mm (bracket) and underlying oedema manifested as subtly increased echogenicity (chevrons), but no drainable fluid collection (a). A comparison with the contralateral (left) breast was performed, which showed normal skin thickness measuring 1 mm (bracket) and normal heterogeneously dense fibroglandular tissue (asterisk) (b). Imaging findings are consistent with right breast mastitis and overlying cellulitis without an underlying abscess. Clinical follow‐up of the right breast erythema and pain with repeat targeted imaging in the setting of worsening symptoms was recommended. Fortunately, this patient's symptoms were fully resolved, and she did not require additional targeted imaging.

Mastitis is present when infection and inflammatory changes progress deeper to involve the breast parenchyma. Ultrasound findings include skin thickening and underlying increased breast echogenicity with associated hyperaemia on colour Doppler. These subtle breast parenchyma changes may make diagnosis difficult, particularly in the setting of heterogeneously dense breast tissue. In such cases, obtaining comparison images of normal parenchyma in an asymptomatic ipsilateral quadrant or the contralateral breast can confirm subtle changes of infection (Figure 3). Additional findings include oedema manifested as interstitial fluid and dilated ducts without a focal fluid collection.^7^ Again, the role of targeted imaging in such cases is to exclude an abscess that has formed deeper in the breast, and commonly, the ultrasound frequency may need to be decreased to appropriately penetrate oedema, fluid and dense breast tissue. Furthermore, harmonic imaging may be used for troubleshooting as there is more tissue contrast between breast lesions and surrounding tissue.^8^

**Figure 4:**
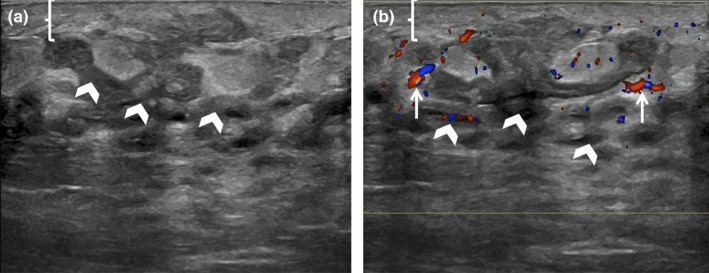
38‐year‐old lactating woman presented with concerns about redness of the left periareolar breast. Greyscale (a) and Color Doppler (b) Ultrasound was performed with a ML6‐15 MHz transducer with the frequency set to 11 MHz, and the images were captured from the skin to the chest wall. Targeted ultrasound evaluation at 6 o'clock 2 cm from the nipple demonstrated mild skin thickening measuring 4 mm (bracket), linear hypoechoic regions of interstitial fluid (chevrons) and associated hypervascularity (arrows). No discrete drainable fluid collection was identified. Constellation of findings was consistent with left breast mastitis with phlegmon. The patient was instructed to continue breastfeeding on a frequent basis, with warm compresses and massage in between, and to complete her 10‐day course of Keflex. Her symptoms were fully resolved, and she did not require a follow‐up ultrasound.

**Figure 5:**
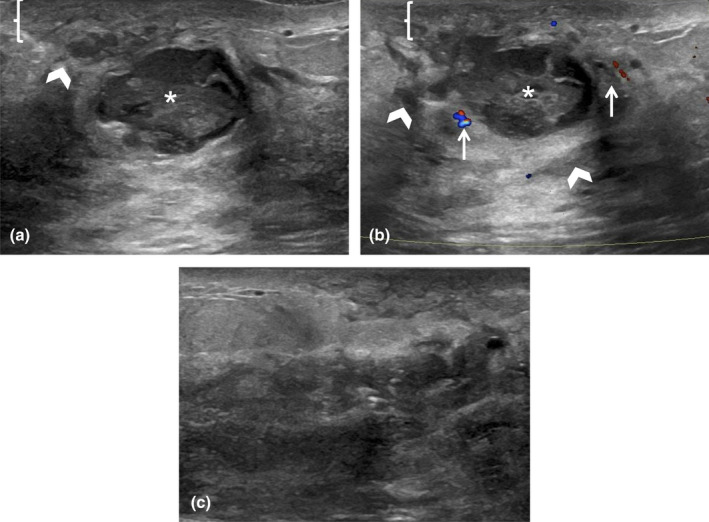
25‐year‐old lactating woman initially felt a 25 mm firm lump in her right breast, which progressively increased in size over 1 week. Ultrasound was performed with an ML6‐15 MHz transducer with the frequency set to 11 MHz, and the images were acquired from the skin to the chest wall. Targeted ultrasound of the redness in the subareolar right breast showed mild skin thickening measuring 4 mm (bracket), oedematous hyperechoic fibroglandular tissue (chevrons) and a discrete complex fluid collection measuring 4.4 cm (asterisk) (a and b). There was also mild surrounding increased vascularity (arrows) (b). Findings were consistent with an abscess, which was confirmed on subsequent percutaneous ultrasound‐guided aspiration, with the near‐complete collapse of the abscess cavity post‐aspiration (c). 15 mL of purulent fluid was aspirated with an 18‐gauge needle and sent to the laboratory to obtain organism sensitivities prior to the initiation of antimicrobial therapy. The patient's symptoms continued and required 2 additional aspirations at 3 and 6 days later.

**Figure 6:**
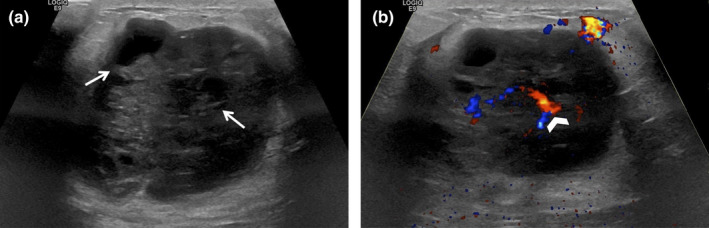
37‐year‐old woman presented to the emergency room with a breast lump, clinically concerning for an abscess. Ultrasound was performed with an ML6‐15 MHz transducer with the frequency set to 9 MHz, and the colour scale was set to 5 cm/s. Targeted ultrasound showed a 4.8 cm heterogeneous hypoechoic mass with internal cystic spaces (arrows) (a). Colour image showed internal vascularity (chevron) but a drainable fluid component was not identified (b). A recommendation was made for further completed diagnostic workup for malignancy in the breast clinic and breast imaging. The evaluation was completed in the breast imaging department 18 days later, where a biopsy was performed confirming invasive ductal carcinoma, with a Nottingham grade of 3/3.

More severe stages of a breast infection include the development of an abscess as the inflammatory response breaks down the infected tissues with the accumulation of bacteria and white blood cells. Only phlegmonous change may be present during the early changes of abscess formation, which on ultrasound presents as breast oedema and non‐contained complex fluid (Figure 4) or non‐drainable thick collections. As the infection continues, a discrete drainable fluid collection with surrounding oedema and increased vascularity may develop (Figure 5). This collection may contain mobile internal echoes and occasional septations.^7^ Thorough ultrasound imaging at the site of clinical symptoms and adjacent breast parenchyma is important to evaluate the extent of the infection for possible intervention and follow‐up. Once an abscess is identified, image‐guided aspiration should be recommended for both diagnostic and therapeutic purposes.^6^ Clinical follow‐up within a week is indicated in these cases and will direct the need for subsequent targeted ultrasound examinations or serial aspirations. If symptoms rapidly progress, patients should be instructed to return to care sooner, or if symptoms are resolving, follow‐up may be extended out further to document complete resolution. Recognising ultrasound features of potential breast malignancy, which may appear as a solid mass, is imperative to avoid misdiagnosis and delayed treatment (Figure 6).^9^ Additionally, malignancies such as inflammatory breast cancers will have a different clinical course. If a patient does not show a clinical response or there is an incomplete response to the appropriate antibiotic therapy within 1–2 weeks, then inflammatory breast cancer should be considered.^10^ As a result, all patients with a breast complaint in an urgent care or after‐hours setting should be advised to have a dedicated imaging evaluation at a breast imaging centre.

**Figure 7:**
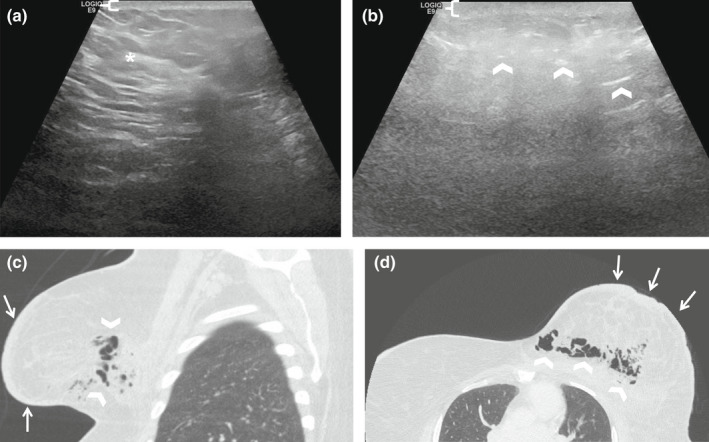
43‐year‐old woman with a history of diabetes and obesity presented with clinical signs of cellulitis and mastitis. Ultrasound was performed with a ML6‐15 MHz transducer with the frequency set to 11 MHz, and the images were acquired from the skin to the chest wall. Targeted ultrasound imaging of the area of clinical concern in the left breast demonstrated mild skin thickening measuring 3 mm (bracket) and mild echogenic breast parenchyma (asterisk) involving the lateral portion of the breast (a). Additional imaging at the area of marked erythema in the medial and inferior breast showed skin thickening measuring 4 mm (bracket) and multiple scattered echogenic foci (chevrons) with posterior dirty shadowing suggestive of subcutaneous air (b). The ultrasound artefact obscured the visualisation of the breast parenchyma posteriorly. Chest CT was immediately performed, revealing gas in the left breast (chevrons) with overlying skin thickening (arrows) as seen on a sagittal image on lung windows (c). Axial chest CT image on lung windows demonstrates air throughout the inner and central left breast extending into the left parasternal subcutaneous tissue (chevrons) with skin thickening (arrows) (d). The on‐call breast surgeon was notified of these critical findings, and the patient was subsequently taken for wide local debridement in the operating room the same day. She required multiple subsequent surgical debridements and ultimately had a mastectomy.

A rare but potentially life‐threatening breast emergency is necrotising fasciitis (NF), most commonly observed in patients with diabetes and obesity.^11^ This soft tissue infection shows rapid progression and results in extensive necrosis of the fascia and subcutaneous tissue, leading to severe systemic sepsis. Targeted ultrasound can identify air as echogenic foci with posterior dirty shadowing (Figure 7).^12^ However, for this particular type of infection, computed tomography (CT) can readily depict the presence of subcutaneous emphysema resulting from the gas‐producing infection and evaluate the extent of disease.^13^

**Figure 8:**
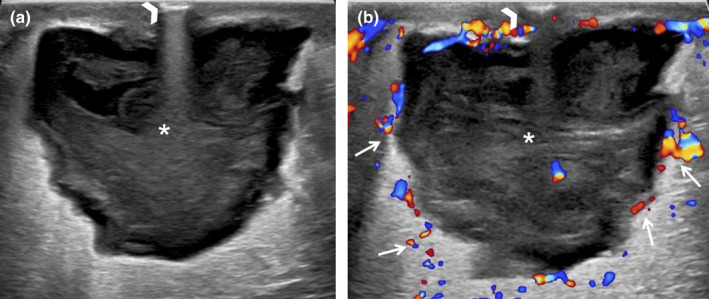
25‐year‐old woman with a right nipple piercing presented with periareolar right breast pain and a lump. Ultrasound evaluation was performed with a L4‐18 MHz transducer with harmonics and the colour gain set to 2.5 cm/s. Greyscale image showed a large complex collection (asterisk) with posterior acoustic through transmission (a). Colour image showed marker peripheral hyperaemia and vascularity (arrows) (b). Both images included linear echogenicity with reverberation artefact from the nipple piercing (chevron).

**Figure 9:**
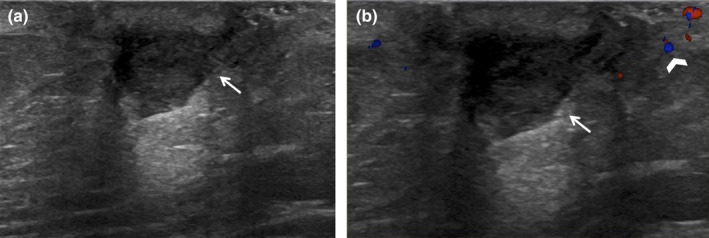
49‐year‐old man with a previous right nipple piercing presented due to a painful palpable lump within the right subareolar region. Ultrasound evaluation was performed with a ML6‐15 MHz transducer at a frequency of 15 mHZ and the colour gain set to 5 cm/s. Greyscale (a) and colour (b) images showed a hypoechoic cystic collection (arrow) directly behind the nipple with minimal surrounding echogenicity and vascularity (chevron).

Body piercing, including nipple piercing, is becoming more common. These adornments can cause various complications including an infection (Figures 8 and 9), allergic reaction or even scarring.^14^ As with any infection, the patient may present with focal breast pain, erythema, swelling and even a palpable mass.^14^, ^15^ These symptoms have been reported to occur anywhere from 2 weeks to 17 months after the piercings have been placed.^14^

Male patients can also present to the emergency department with complaints of a possible breast infection. However, unlike with women, infections in the male breast are typically a result of trauma such as piercings (Figure 9).^16^

### Iatrogenic haemorrhage

**Figure 10:**
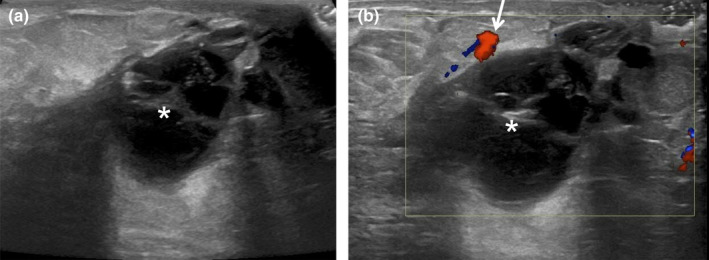
73‐year‐old woman with increased pain and bruising after an ultrasound‐guided biopsy, which was performed with a 9‐gauge vacuum‐assisted device. Ultrasound of the right breast at 9 o'clock 4 cm from the nipple shows a complex cystic collection measuring 3 cm (asterisk), consistent with a post‐biopsy haematoma (a). Colour Doppler imaging shows a single prominent adjacent vessel (arrow) without active bleeding or pseudoaneurysm (b). The breast/chest was wrapped in a compression bandage and instructed to limit activity and return to care if symptoms worsened.

**Figure 11:**
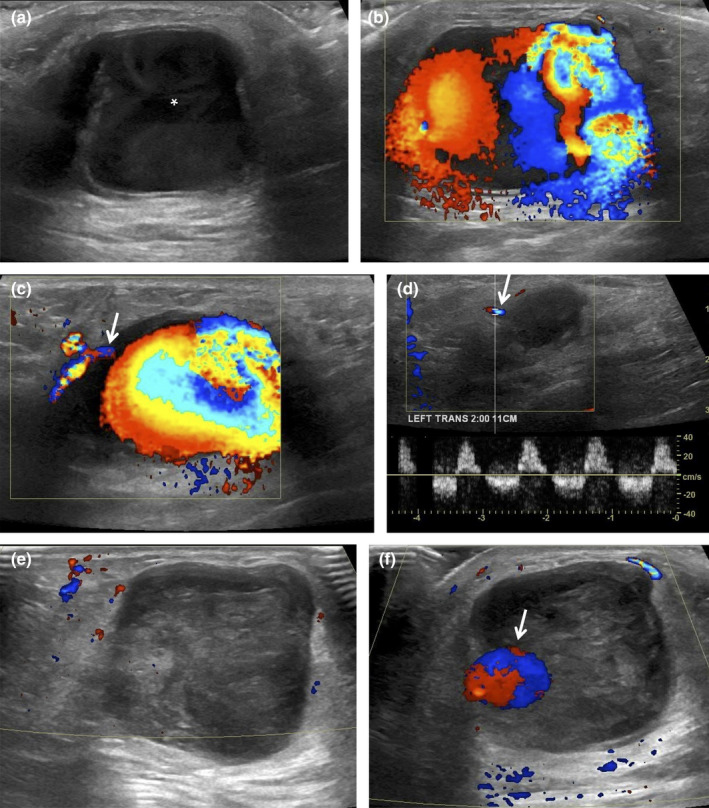
75‐year‐old woman with a biopsy performed at an outside institution with a 9‐gauge vacuum‐assisted device. This was a lump that was enlarging after the biopsy site on physical examination. Targeted ultrasound evaluation at 2 o'clock 11 cm from the nipple, adjacent to the biopsy‐proven malignancy, demonstrated a (a) 3.0 cm heterogeneous fluid collection (asterisk) with mobile swirling echoes observed real time, and (b) colour Doppler showed a yin–yang colour pattern in the collection, with a (c) single feeding vessel (arrow). (d) Spectral Doppler of the feeding vessel neck revealed a to‐and‐fro waveform, consistent with a pseudoaneurysm. With a narrow neck, this pseudoaneurysm was amenable to percutaneous thrombin injection (1 mL of 1000 IU/mL thrombin). (e) Immediate post‐thrombin injection image showed complete thrombosis of the PsA. (f) A follow‐up ultrasound 1 week later showed that most of the PsA had thrombosed with a small residual pseudoaneurysm (arrow). Repeat thrombin injection was performed with a total of 1.7 mL of thrombin injected at the pseudoaneurysmal neck and other small contributors to the pseudoaneurysm. No further intervention with thrombin was required.

**Figure 12:**
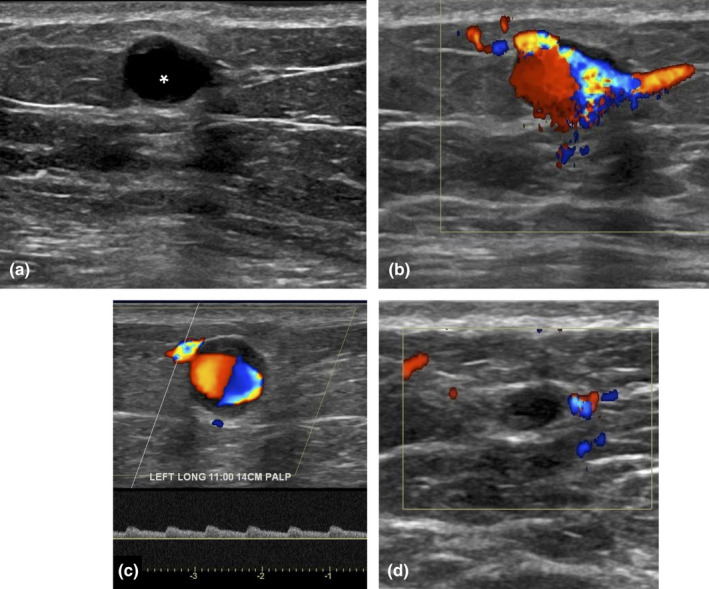
41‐year‐old woman with no prior history of trauma or intervention presented with a palpable left breast lump. Targeted ultrasound showed a 1.0 cm anechoic cystic structure (asterisk) at 11 o'clock 14 cm from the nipple (a), with turbulent flow colour with some mural thrombus (b). Spectral Doppler of the feeding vessel demonstrates a low resistive arterial waveform (c) and to‐and‐fro flow was not seen. This did not have the typical appearance of a pseudoaneurysm, but rather a true aneurysm. Given this, thrombin injection was deferred, and surgery consultation was recommended. Breast and vascular surgery agreed on short‐interval follow‐up given the mild symptoms. This patient returned 5 months later, and the true aneurysm was smaller in size and had completely thrombosed (d).

**Figure 13:**
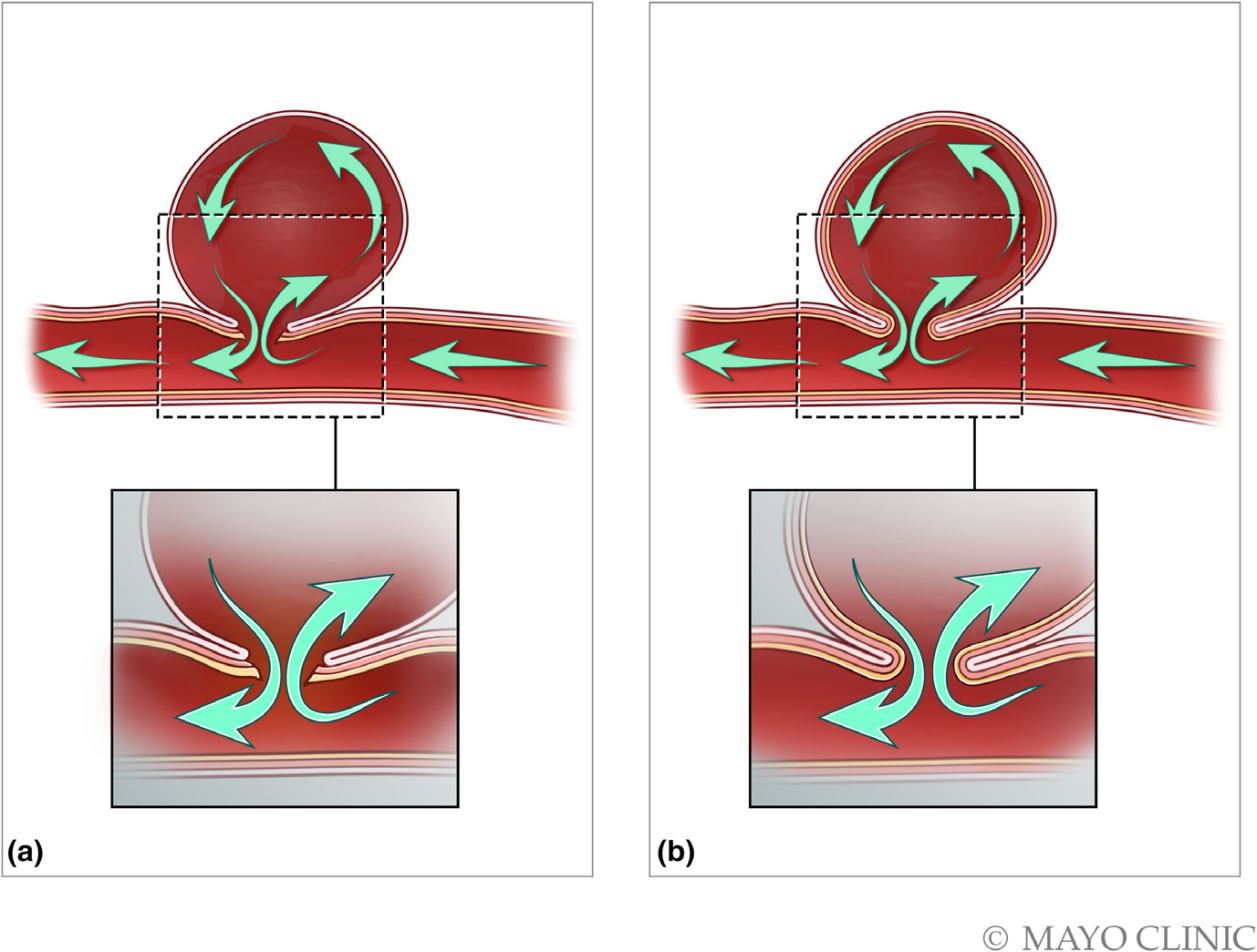
Artist comparison of a PsA *ve* *rsus* a true saccular aneurysm. (a) Illustration of the pseudoaneurysm, which does not contain all 3 layers of the arterial wall. (b) In comparison, a true saccular aneurysm involves the intima, media and adventitia. Source: Used with permission of Mayo Foundation for Medical Education and Research, all rights reserved.

Haematoma is the most common post‐procedural complication. Bleeding typically occurs immediately or within 24 h of a biopsy. The goal for post‐procedural imaging in this setting should be to determine whether there is active bleeding from a vessel with an expanding haematoma or a stable thrombosed haematoma (Figure 10).^17^ If active bleeding is present, dedicated images to evaluate a pseudoaneurysm (PsA) with colour Doppler imaging to evaluate a typical yin–yang sign are important. If a PsA is present, images of the neck, including size and spectral Doppler looking for characteristic to‐and‐fro or bidirectional flow, can assist the interventionalist (Figure 11). Based on the neck size and shape, it is possible to determine whether the percutaneous thrombin injection can be used to treat pseudoaneurysm.^18^ A narrow neck is optimal for consideration of either compression or thrombin injection. Furthermore, a PsA should be differentiated from a true aneurysm, which involves all three layers of the blood vessel (Figures 12 and 13).

**Figure 14:**
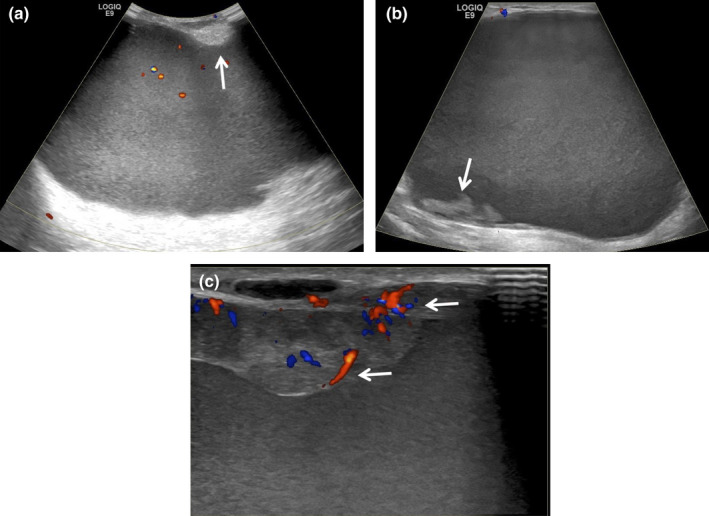
56‐year‐old woman presented to the emergency department due to uncontrolled right breast bleeding. Initial overnight ultrasound images performed with a C1‐6 MHz transducer set to a frequency of 5 MHz by the general sonographer showed a large unilocular cystic lesion with homogeneous internal low‐level echoes and mural nodules (arrow) (a). Repeat imaging 1 day later was done prior to biopsy using a ML6‐15 MHz transducer with the frequency set to 9 MHz in the breast imaging department confirming the large unilocular cystic lesion with irregular margins and homogeneous internal low‐level echoes (b). With reduced manual compression from the transducer and the colour scale set to 5 cm/s, internal vascularity was identified in the mural nodules (arrow) (c). Ultrasound‐guided aspiration of the fluid component and biopsy of the solid component was performed revealing intracystic papillary carcinoma with a low nuclear grade associated with a haematoma.

Very rarely patients present with ‘spontaneous’ bleeding from the breast. A physical examination will often reveal an associated wound or underlying breast mass suspicious of malignancy.^19^ The neoangiogenesis that occurs with malignancies results in friable vessels, which are prone to haemorrhage.^7^, ^20^ Papillary tumours have vascular stalks, which, when severed, can lead to repeated bleeding into a cystic component. Ultrasound will show a mixed solid and cystic mass.^21^ Identifying flow in the solid components of these lesions can aid in an accurate diagnosis and often requires lightening the manual pressure and reducing the colour scale to detect colour flow (Figure 14). Non‐emergent next‐day sampling of the solid portion should be completed by a dedicated breast imaging team.

### Trauma

**Figure 15:**
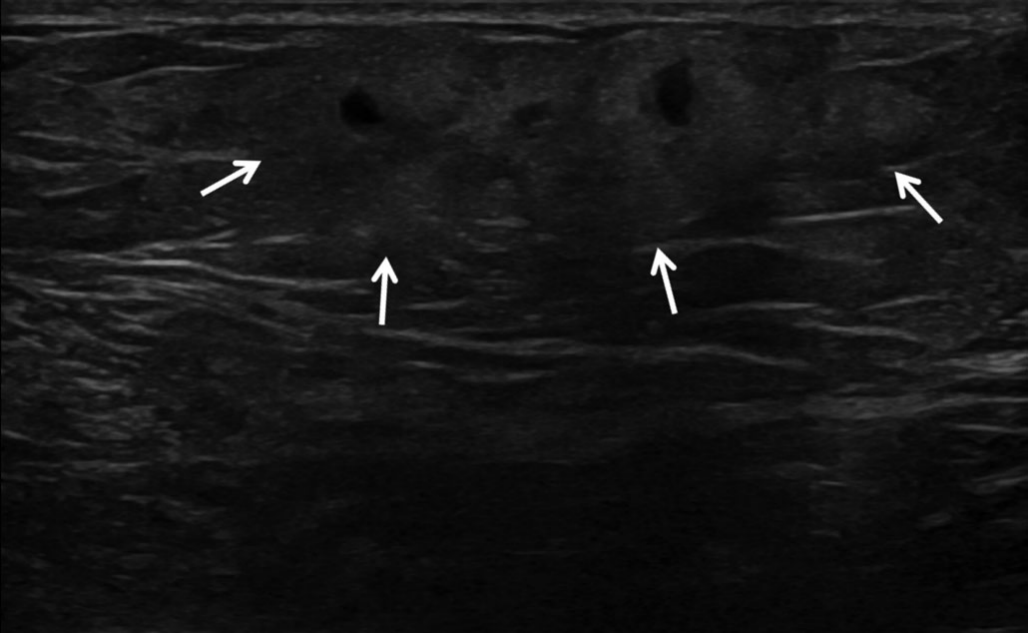
89‐year‐old woman with trauma to the left breast after a fall, which resulted in extensive bruising and multiple palpable lumps. Targeted ultrasound was performed with a ML6‐15 MHz transducer with the frequency set to 15 MHz and showed a 3 cm area of superficial hyperechogenicity (arrows) containing a few small cystic spaces, most consistent with acute fat necrosis.

**Figure 16:**
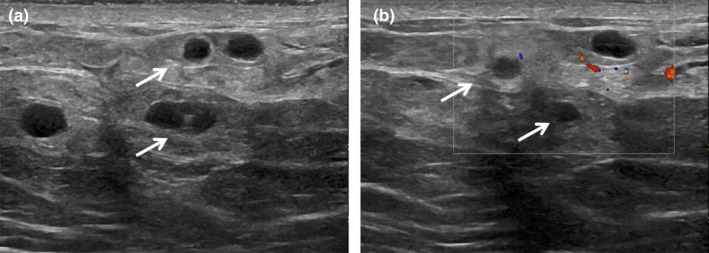
37‐year‐old woman presented to the emergency department with left breast pain. An electronic medical chart review showed a history of breast trauma a month prior. Ultrasound evaluation to exclude a fluid collection carried out by the general on‐call sonographer was performed with a ML6‐15 MHz transducer with the frequency set to 13 MHz. Greyscale (a) and colour doppler (b) images demonstrated multiple round and oval circumscribed masses (arrows), which have the appearance of subacute fat necrosis.

Blunt force trauma to the breast is commonly seen in motor vehicle accidents and falls, which can result in haematoma formation and subsequent fat necrosis.^22^ In the acute setting, ultrasound of the affected area will show predominantly hyperechoic breast tissue due to breast oedema (Figure 15). In the subacute phase, multiple cystic spaces develop with or without surrounding echogenic fat (Figure 16). As there is evolution of the fat necrosis, the sonographic findings may fully resolve the patient's symptoms, or the cystic components may become discrete oil cysts, which can be calcified over time.^23^, ^24^

**Figure 17:**
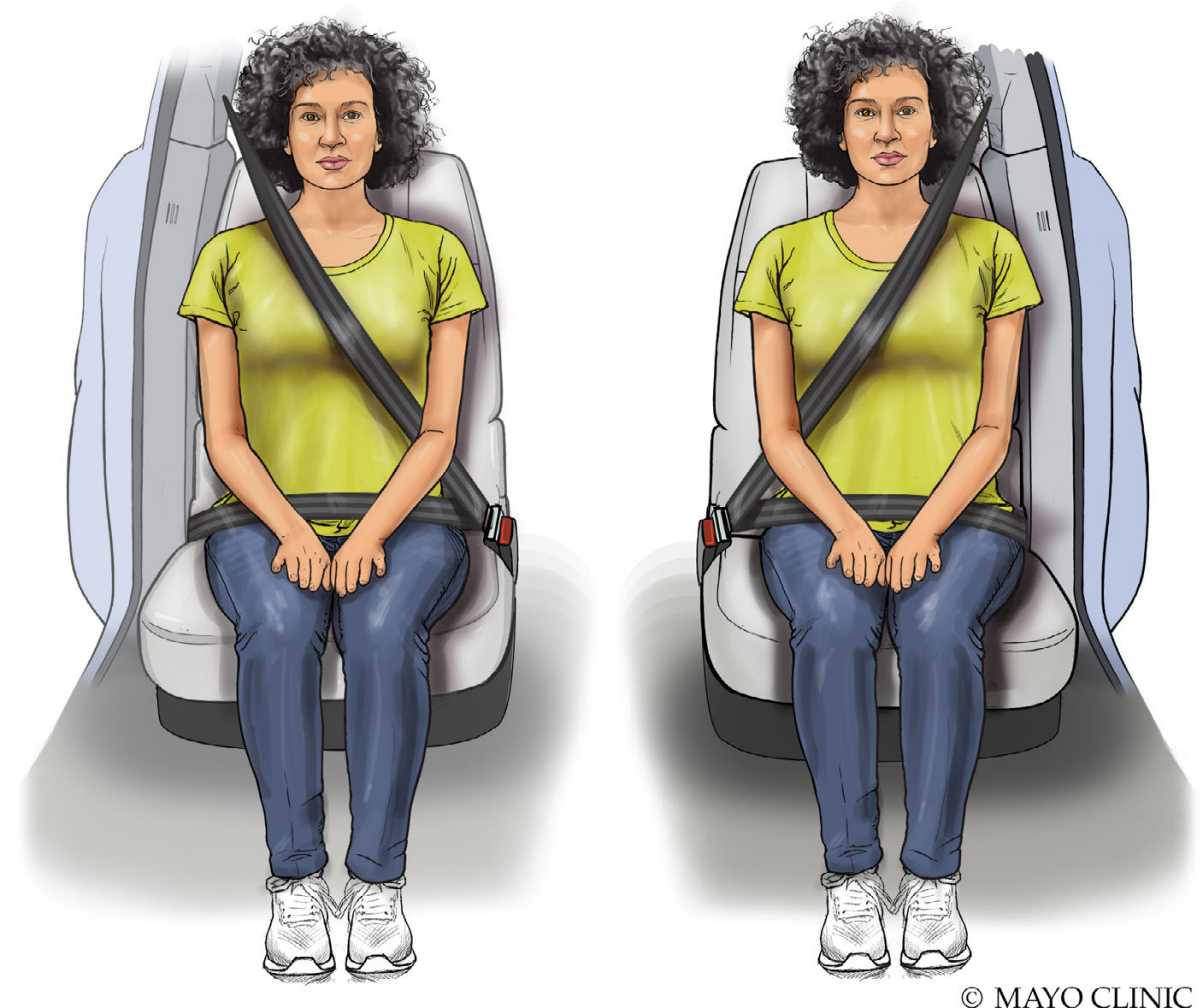
Illustration of how seat belt positioning affects the location of the injury depending on if the patient is seated on the driver's side or the passenger's side of the vehicle. The orientation of the seat belt will determine the location and pattern of injury. Knowing the non‐ductal distribution helps evaluate sites of injury during the initial evaluation. However, this distribution plays an important role in the subsequent non‐emergent follow‐up for the patient. Source: Used with permission of Mayo Foundation for Medical Education and Research, all rights reserved.

**Figure 18:**
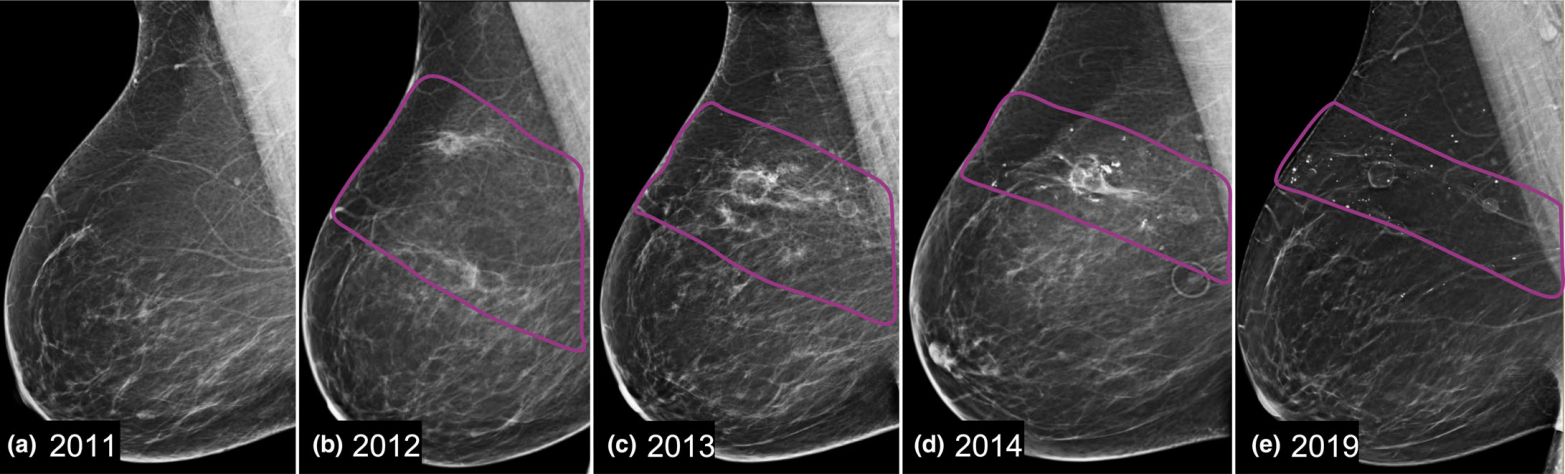
Serial right breast MLO mammogram images (a–e) in a patient with a seat belt injury after a motor vehicle collision where the patient was in the driver seat in 2011 (a). In 2012 the classic described non‐ducal band‐like pattern and location of fat necrosis in the upper right breast was seen (a). An asymmetry developed in 2012 (b), with initial diagnostic evaluation attributing the findings to fat necrosis in the setting of trauma. Subsequent images (c–e) show the normal evolution of fat necrosis into oil cysts in a band‐like orientation, which slowly retracts over several years. Abbreviation: MLO, Mediolateral Oblique.

Seat belt injuries will have a discrete pattern and location depending on if the patient is sitting in the driver's seat or the passenger's seat (Figure 17).^25^ The orientation of the seat belt is such that the appearance of the injury on subsequent mammograms will be in a non‐ductal distribution (Figure 18). Imaging findings will vary depending on the timing of the injury and subsequent patient presentation for care. Acute presentation may show increased echogenicity due to trauma or even a developing haematoma if the injury is severe, whereas a delayed presentation may show the later stages of fat necrosis.^15^, ^26^

### Ethics approval

This review was approved by the Mayo Clinic IRB.

## Conclusions

There are very few breast lesions that are considered a true breast imaging emergency. However, patients often present to the emergency department or urgent care for infection, bleeding and trauma. Knowing how to image and diagnose these cases correctly is important in guiding referring providers with the appropriate recommendations and follow‐up, ultimately providing the patient with the appropriate care and avoiding missing breast cancers.

## Authorship statement

The authors have nothing to disclose. Dr. Asha Bhatt contributed to the idea and concept of the manuscript. All authors equally contributed to the original draft and review of the manuscript, and are in agreement with the content of the submitted manuscript.

## Funding

No funding information is provided.

## Conflict of Interest

None declared.

## Author contributions

**Asha A. Bhatt:** Conceptualization (lead); resources (lead); writing – original draft (lead); writing – review and editing (lead). **Genevieve A Woodard:** Resources (equal); writing – original draft (equal); writing – review and editing (equal). **Christine U Lee:** Conceptualization (supporting); writing – original draft (equal); writing – review and editing (equal). **Gina K Hesley:** Writing – original draft (equal); writing – review and editing (equal).
